# A personalised approach for identifying disease-relevant pathways in heterogeneous diseases

**DOI:** 10.1038/s41540-020-0130-3

**Published:** 2020-06-09

**Authors:** Juhi Somani, Siddharth Ramchandran, Harri Lähdesmäki

**Affiliations:** Department of Computer Science, Aalto University, 02150 Espoo, Finland

**Keywords:** Computer modelling, Time series, Robustness, Nonlinear dynamics, Software

## Abstract

Numerous time-course gene expression datasets have been generated for studying the biological dynamics that drive disease progression; and nearly as many methods have been proposed to analyse them. However, barely any method exists that can appropriately model time-course data while accounting for heterogeneity that entails many complex diseases. Most methods manage to fulfil either one of those qualities, but not both. The lack of appropriate methods hinders our capability of understanding the disease process and pursuing preventive treatments. We present a method that models time-course data in a personalised manner using Gaussian processes in order to identify differentially expressed genes (DEGs); and combines the DEG lists on a pathway-level using a permutation-based empirical hypothesis testing in order to overcome gene-level variability and inconsistencies prevalent to datasets from heterogenous diseases. Our method can be applied to study the time-course dynamics, as well as specific time-windows of heterogeneous diseases. We apply our personalised approach on three longitudinal type 1 diabetes (T1D) datasets, where the first two are used to determine perturbations taking place during early prognosis of the disease, as well as in time-windows before autoantibody positivity and T1D diagnosis; and the third is used to assess the generalisability of our method. By comparing to non-personalised methods, we demonstrate that our approach is biologically motivated and can reveal more insights into progression of heterogeneous diseases. With its robust capabilities of identifying disease-relevant pathways, our approach could be useful for predicting events in the progression of heterogeneous diseases and even for biomarker identification.

## Introduction

With the increasing affordability of high-throughput technologies, such as microarray and RNA sequencing, genome-wide time-course gene expression data has become one of the most abundant and routinely analysed type of data^1^ for studying and understanding the molecular mechanisms underlying various complex diseases^2^. Encapsulating a wealth of information regarding the prolonged or transient expressions of a large set of activated genes^1^, time-course data also helps us understand and model the (multidimensional) dynamics of complex biological systems or phenomena, such as disease progression^1,3,4^. It offers us the possibility of deciphering the underlying pathophysiologies and systematic evolutions of human diseases^3^. A prominent goal in such studies has been to identify genes whose expression levels systematically differ between a case (e.g., disease) and a control (e.g., healthy) group, and can be classified as biomarkers for diagnosis and prognosis of the disease.

For over a decade, various methods have been introduced for modelling time-course data to identify differentially expressed genes (DEGs). Nonetheless, modelling, interpreting and validating the gene expression patterns are continually met with major challenges. The challenges can be largely classified into two categories: (i) robustly modelling the dynamics of time-course data and (ii) accounting for the heterogeneity of complex diseases.

Many methods have been proposed that deal with the most prominent limitations of modelling gene expression time-course data. Some such limitations include non-uniform sampling^1,5^, too few sampling times, missing time points, few or no replicates^5^, autocorrelation between successive time points^5,6^, and high-dimensionality with small sample sizes^4^. Some methods simplify the modelling task by disregarding the dynamic nature and making the expression profiles “coarse-grained”^4^, such as cross-sectional analysis (i.e., direct time point-wise comparison of samples) ^7^ and simplification strategies^4,8,9^. However, these methods are suboptimal. Interpolation methods, such as linear^10^ and B-spline (cubic spline)^7,11,12^, have been one of the first methods to be attempted for modelling the dynamics of longitudinal data and using them for estimating gene expression levels at unobserved time points^5,6,7^. Even though they incorporate the continuous nature of the data, they may be subject to issues, such as overfitting. In fact, B-spline-based methods require more than ten time points to produce reliable results^5,6^, which makes it unsuitable for applications in many biological studies^4^.

Recently, linear mixed models (LMMs) and Gaussian processes (GPs) have become popular choices for time-course data modelling due to their ability of modelling the correlational structure of the data^13,14,15^; efficiently handling biological replicates, while accounting for subject-specific variability; including time-invariant and time-varying covariates; and determining the trends over time, as well as taking into account the correlation that exists between successive measurements^16^. Moreover, GP models offer a robust way of estimating missing or unobserved values by providing confidence intervals along the estimated curves of gene expression^16^. GP models can be used to identify differential expression between multiple conditions^17^ or handle general experimental designs^18^. They can also be designed to be robust to outliers and employ flexible model basis^19^. GPs capture the underlying true signal and embedded noise in a time-course gene-expression data in a non-linear manner, without imposing strong modelling assumptions. In addition to answering whether a gene is differentially expressed across the whole time-course, GP models have also been successfully applied for determining specific time-windows when a gene is DE even when no or few observations are made in that time-window^19,20,21^.

The traditional applications of these methods detect genes that exhibit different expression levels between a case and a control group (DEGs) across the whole study population. Unfortunately, in the case of heterogeneous data from complex diseases, only a few genes are usually found to be DE across all or most cases because different genes with similar functionalities may be found to be perturbed across cases, thus justifying the gene-level variability at a functional or pathway-level^2^. In fact, gene-level results from similar studies of heterogeneous diseases, such as cancers^22,23^, asthma, Huntington’s diseases^2^, rheumatoid arthritis, type 2 diabetes, schizophrenia^24^, and Parkinson’s disease^2,24^, have often been found to be inconsistent. They show distressingly little overlap between similar studies of the same disease^2,22,25,26^. Due to these challenges, many methods that summarise the results on a pathway-level have been developed, where the genes are unified under biological themes that aid in a functional understanding of the results. This can be further improved by developing personalised approaches for identifying enriched or disrupted pathways in complex diseases. Here, personalised approach refers to such methods that do not assume that changes are consistent across all study subjects but instead they identify biomarkers for each subject, e.g., by analysing each case-control pair separately; and a pathway is an overarching term for a group of genes unified under biological themes and are also referred to as gene sets in Subramanian et al.^25^.

Menche et al.^2^ introduced a framework for personalised gene expression analysis, where personalised perturbation profiles (PEEPs) are constructed per case subject by calculating a *z*-score with reference to the control group and considering any gene with a *z*-score above an optimised threshold to be part of the PEEP. Using a combinatorial model on the PEEPs, they strive to identify a single pool of disease-associated genes that can be used to accurately predict the disease status of each subject. The method of Menche et al.^2^ thus accounts for heterogeneity. However, it is not directly suitable for modelling time-course data.

Pathway (gene set) enrichment analyses, such as Fisher’s exact test and GSEA^25^, are commonly applied to the gene-level results in order to obtain an understanding of the results at the level of biological processes. Several specialised methods have also been proposed for pathway-level analysis with two groups, such as module map^22^, CORGs^27^, Pathifier^23^, SPCA^28^, and PARADIGM^29^. However, only a few can be applied directly to time-course experiments. One such method is the unified statistical model for analysing time-course experiments at the pathway-level using linear mixed effects models^30^. This method directly identifies significant pathways expressed over time by using random effects to model the heterogeneous correlations between the genes in the pathway, as well as other fixed and random effects. Unfortunately, these methods do not apply a personalised approach for the modelling.

In this paper, we propose a method that models the time-course data in a personalised manner using Gaussian processes and combines the lists of DEGs on a pathway-level. Our method assumes an experimental design where each case subject is matched with a carefully chosen control subject, and the method uses a robust yet efficient method to detect DE genes for each individual with respect to the matched control. Individual-specific gene-level results are summarised at pathway-level using a permutation-based empirical hypothesis testing that is tailored for personalised DE analysis. To study expression changes associated with particular time periods, such as time before disease onset, we also extend the method to detect DEGs in specific time-windows. This method can be applied to longitudinal case-control data from different technologies, such as gene expression microarray, RNA sequencing and polymerase chain reaction (PCR), and to a variety of omics data types. To our knowledge, there are no competing methods to our proposed method.

We applied this method to largely two type 1 diabetes (T1D) microarray datasets from Kallionpää et al.^31^. There is growing evidence that T1D is a genetically heterogeneous disease^32–34^. Therefore, in order to gain a robust understanding of the molecular mechanisms underlying this complex and heterogeneous disease, one needs to apply a personalised approach on a pathway-level like the one presented here. We report disruptions in pathways during the early progression of T1D (time-course analysis), as well as in the 6 months windows before seroconversion (autoantibody positivity) and clinical diagnosis of T1D. Seroconversion is the time of autoantibody presentation in T1D susceptible individuals and represents the earliest (currently known) signs of disease progression. However, clinical diagnosis of T1D is established at a very late stage of the disease when insulitis has persisted over a long period of time^35,36^; ~80–90% of *β*-cells have been destroyed; and hyperglycaemia is achieved^31,35^. Therefore, identifying relevant perturbations at different stages of the disease can help in monitoring and perhaps predicting the significant events in the disease progression. Our personalised approach was able to identify various disease-relevant and interesting pathways from all three analyses, including those that illustrate the intrinsic mechanisms of disease progression. We also compared the results of the proposed personalised approach with those of a population-wide method, the original results from Kallionpää et al.^31^ and also a third T1D dataset from Ferreira et al.^37^. This method can be applied to other heterogeneous diseases with a similar experimental design and also extended to non-paired case-control datasets.

## Results

### Overview of our personalised GP regression and pathway detection method

**Fig. 1:**
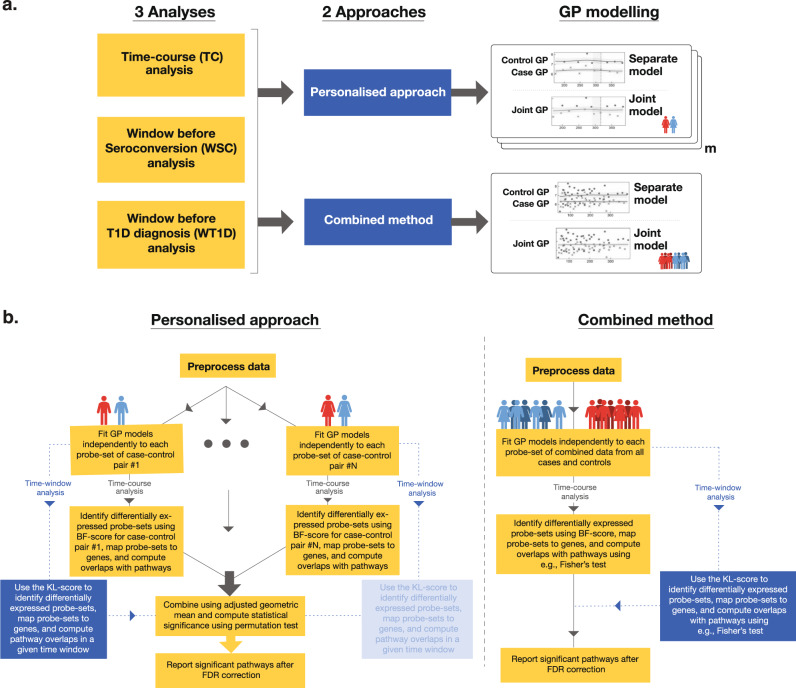
A schematic overview of our personalised approach as well as the combined method (a population-wide approach). **a** Summary of the analyses performed and approaches used in this study as well as a depiction of the *separate* and *joint* models used for GP modelling in the analyses. Here, *m* is the number of case-control pairs. **b** A schematic illustration of identifying differentially expressed genes (DEGs) and significant pathways using the personalised approach and the combined method. In the personalised approach, the DEGs are identified independently for each case-control pair and summarized on a pathway-level, whereas in the combined method, DEGs are identified by comparing all cases to all controls.

In this paper, we present a personalised approach for identifying enriched pathways given time-course observations from multiple two-sample (matched case-control) pairs. We apply our method on gene expression microarray datasets with varying number of case/control observations per pair and uneven sampling times. We performed three types of analyses using *Datasets 1* and *2* described in section on Data: early disease progression time-course (TC) analysis across the whole study period, time-series analysis within a window before seroconversion (WSC), and time-series analysis within a window before T1D diagnosis (WT1D). We compared the results obtained using our proposed personalised approach in each of the three analyses with those obtained using a combined (non-personalised) method. Fig. 1 gives a high-level overview of our analyses and highlights the differences between the two approaches discussed in this paper.

In our personalised approach, we examine each feature (i.e., probe-set or gene) from each case-control pair separately by fitting two models, *joint* and *separate*. In the *joint* model, a GP regression is fit to all samples from a case-control pair together (corresponds to the null hypothesis), whereas in the *separate* model, GP regressions are fit to cases and controls separately (corresponds to the alternative hypothesis). We identified the DE features for each case-control pair separately by quantifying the fitting of each model using BF-scores and KL-scores in time-course and time-window analyses, respectively (see Eqs. (6) and (13)). A feature was classified as DE when, in TC analysis, the BF-score was above 4 and, in time-window analyses, the KL-score was above 250. In case of probe-sets, we mapped the DE probe-sets from each pair to their corresponding gene names and performed pathway-level analysis on DE genes. Subsequently, we proceeded to obtain an enrichment score (over all case-control pairs) for each pathway from MSigDB^25,38^ using the metric in Eq. (15). This was followed by a permutation test for identifying a set of enriched pathways.

Our personalised approach is significantly different from the combined method where we compute the associated BF-scores and KL-scores per feature by pooling together all the cases and all the controls to form a set of combined cases and controls (assuming gene expression difference is homogeneous across the whole study population). The enriched pathways are then identified using a standard one-sided Fisher’s exact test.

A detailed description of the personalised and combined methods can be found in Methods section.

### Data

In this study, two T1D time-course gene expression datasets, (*Datasets 1* and *2*) published by Kallionpää et al.^31^, were primarily analysed to understand pathway-level disruptions in T1D (TC, WSC, and WT1D analyses). We also perform TC analysis using our personalised approach on a different T1D dataset, (*Dataset 3*) published by Ferreira et al.^37^, to assess the generalisability of our method and results. All three datasets were generated by hybridising total mRNA extracted from venous blood cells on microarrays (Affymetrix U219 arrays in Kallionpää et al.^31^ and Affymetrix Human Gene 1.1 ST arrays in Ferreira et al.^37^). Kallionpää et al.^31^ matched each case individual to a healthy control individual based on confounding factors, such as date and place of birth, gender and HLA risk class, and hybridised samples in batches based the pairing. Similarly, we paired cases and controls from Ferreira et al.^37^ based on time of birth, gender and sampling ages. The raw datasets were downloaded from public databases (see section on Data availability) and preprocessed using *affy*-packages and *oligo*-packages in *R*. More details are given in the Supplementary Notes and the respective articles.

*Dataset 1* comprised of 80 samples from six case-control pairs (43 case and 37 control samples) chosen from the sample series of *seroconverted progressors*, such that each pair was sampled before and after seroconversion of the case. *Dataset 2* comprised of 188 samples from 15 case-control pairs (103 case and 85 control samples) chosen from the sample series of *T1D progressors*, such that each pair was sampled starting after seroconversion and till at least one month before T1D diagnosis of the case. Therefore, *Dataset 1* was used for the early disease progression time-course (TC) and window before seroconversion (WSC) analyses; whereas, *Dataset 2* was used for the window before T1D (WT1D) analysis. *Dataset 3* comprised of 126 samples from 9 case-control pairs (79 case and 47 control samples), such that each pair was sampled before and after seroconversion of the case and each individual was sampled at least at three time points. All individuals chosen in each dataset consisted of 3 to 12 longitudinal samples each.

For pathway information, we used the Molecular Signatures Database (MSigDB, v6.1), which is a collection of annotated gene sets^25,38^. We performed pathway-level analyses using 16808 (of 17786) pathways from the collection.

### Identifying differentially expressed genes

Differentially expressed genes (DEGs) were identified in a direction-agnostic manner for pathway-level evaluation in all three analyses using both the personalised and combined approaches. In *Datasets 1* and *2*, the personalised approach resulted in an average of 895, 1127 and 1677 genes DE in the TC, WSC and WT1D analyses, respectively. On average, 14% (TC: 13%, WSC: 13%, WT1D: 17%; obtained by dividing the average number of overlapping DEGs between all pairs with the average number of DEGs in each pair) of the DEGs overlapped between DEG lists of each case-control pair in the three analyses, thereby demonstrating heterogeneity among case-control pairs. The combined method resulted in 436, 234, and 563 genes as DE in the TC, WSC and WT1D analyses, respectively. The overlap of DEGs between the two approaches was significant in all analyses (*p*-value < 0.05 using Fisher’s exact test).

**Fig. 2:**
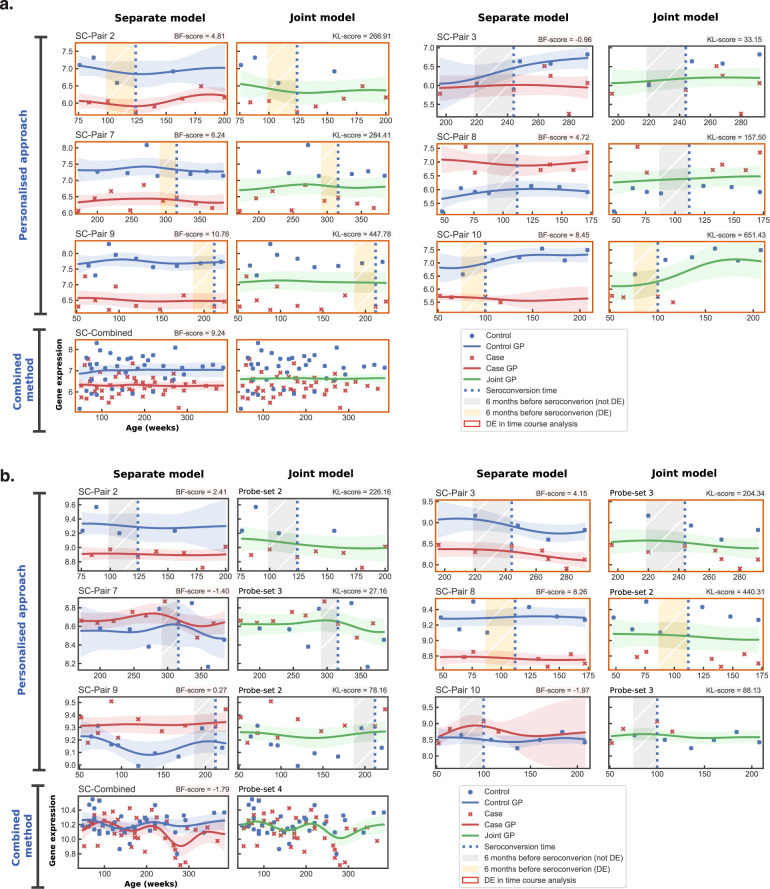
Gene expression plots of two genes visualising the GP model fittings of the *separate* and *joint* models for the six case-control pairs from *Dataset 1.* **a** Gene expression plots for *PTPRN2*. All profiles belong to the same probe-set as all pairs, including the combined method, which identified the same probe-set to have the largest BF-score for *PTPRN2*. **b** Gene expression plots for *HSPD1*. The probe-set information is marked for each pair since the profiles identified different probe-sets to have the largest BF-score for *HSPD1*. Shaded area around each GP regression represents the 90% confidence interval of the fitting. A red border around a plot signifies differential expression (DE) in the time-course analysis and an orange shaded window signifies DE in the time-window analysis. Here, pairs from *Dataset 1* are prefixed with ‘SC-’.

The personalised approach accounts for the heterogeneity between the pairs in time-course and time-window analyses. Firstly, if probe-sets are used, the differential expression of a gene in a case-control pair could be attributed to any of its probe-sets regardless of the probe-set expressed in other pairs. Secondly, the dynamics of gene expression and even the direction of regulation of a DEG is allowed to vary from one case-control pair to another. Although unclear, certain genes may be behaving inconsistently across individuals due to the presence of certain other genes; or any deviation, regardless of the direction, could result in disease-associated perturbation possibly because of the mechanism of regulating the pathway^2^. Thirdly, even a gene that is not differentially regulated in most of the case-control pairs can be relevant on the pathway-level. Finally, the GP modelling was able to robustly interpolate over unobserved time points (see sections on robustness analysis in Methods and Supplementary Methods), which was especially important in time-window analyses where sometimes only a few or no samples were available for determining differential expression, as can be seen in Figs. 2a and b, as well as Supplementary Figs. 1 and 2.

The combined method, on the other hand, is more stringent when identifying DEGs in time-course and time-window analyses. For a gene to be identified as DE using this method, a feature is usually required to be DE in almost all of the pairs. Furthermore, if a gene exhibits different temporal expression dynamics or is regulated in opposite directions in different pairs, this model is unlikely to identify it as differentially expressed.

To illustrate the above-mentioned traits, the expression of the genes encoding the only two autoantigens that were differentially expressed in the TC and WSC analyses, *PTPRN2* and *HSPD1*, from T1D pathway are shown in Figs. 2a and b. *PTPRN2* encodes a major islet autoantigen in T1D, which plays an important role in insulin secretion in response to glucose stimuli by accumulating normal levels of insulin-containing vesicles and preventing its degradation^39^. *HSPD1* is considered a pro-apoptotic or anti-apoptotic regulator of apoptosis, depending on the circumstances^40^, whose high-levels have been associated with diabetes, as well as increased expression of inflammatory genes and release of pro-inflammatory cytokines^41,42^. In the TC analysis of *Dataset 1* using the personalised approach, case-control pairs 2, 7, 9, and 10 differentially downregulated only the *PTPRN2* gene; pair 3 downregulated only the *HSPD1* gene; and pair 8 downregulated *HSPD1*, but upregulated *PTPRN2*. Here, the pairs regulating *HSPD1* differentially express different probe-sets of the gene, whereas all pairs regulating *PTPRN2* differentially express the same probe-set. However, pair 8 upregulated *PTPRN2* when other pairs downregulated it. Coincidentally, pair 8 is the only pair that expressed both *PTPRN2* and *HSPD1* in this data and it downregulated *HSPD1* while upregulating *PTPRN2*, which may indicate correlation between the two. On the other hand, the combined method found significance only in the *PTPRN2* gene since 5 of 6 case-control pairs differentially expressed the same probe-set. Moreover, Supplementary Figs. 1 and 2 show two examples, *HLA_DPB1* (probe-set: 11760799_x_at) and *IRF5* (probe-set: 11726687_a_at), where the case-control pairs regulate the genes in inconsistent directions. Here, the combined method identifies *HLA_DPB1* as DE, whereas *IRF5* is classified as insignificant. The personalised approach, however, identifies both of these genes as significant in all pairs.

### Combined method vs. personalised approach

Using the combined method, 52, 10, and 80 pathways were found to be significantly enriched with FDR < 0.1 in the TC and WSC analyses of *Dataset 1* and WT1D analysis of *Dataset 2*, respectively. Similarly, 124, 307, and 2550 pathways were found to be significantly enriched with FDR < 0.1 in the TC, WSC and WT1D analyses, respectively, using the personalised approach (Table 1, Supplementary Data). Of these, 12, 1, and 38 enriched pathways overlapped between the two approaches in the TC, WSC and WT1D analyses, respectively, which was found to be a significant amount (Fisher’s combined *p*-value < 0.0001 obtained from p-values determined using Fisher’s exact test) (Table 2). Nonetheless, the combined method was unable to identify most of the immunologically interesting and disease-relevant pathways in all three analyses that were identified using the personalised approach.

**Table 1:** Number of pathways identified as enriched (FDR < 0.1) in TC, WSC and WT1D analyses using the personalised approach, combined method and gene-level results from Kallionpää et al.^31^.

Approaches/Analyses	TC	WSC	WT1D
Combined method	52	10	80
Personalised approach	124	307	2550
Kallionpää et al.	386	432	824

**Table 2:** Number of enriched pathways overlapping between different approaches in the TC, WSC and WT1D analyses (rows marked as ‘count’). A *p*-value, determined using Fisher’s exact test, is also given for each overlap to show its significance (rows marked as ‘*p*-value’), where NS refers to ‘not significant’ *p*-values (i.e., *p*-value > 0.05). Fisher’s combined *p*-value over all analyses per comparison is given in the last column.

Overlaps/analyses		TC	WSC	WT1D	Fisher’s combined *p*-value
Combined method vs. personalised approach	Count	12	1	38	<0.0001
	*p*-value	<0.0001	NS	<0.0001	
Kallionpää et al. vs. personalised approach	Count	29	60	507	<0.0001
	*p*-value	<0.0001	<0.0001	<0.0001	
Kallionpää et al. vs. combined method	Count	5	0	5	NS
	*p*-value	<0.01	NS	NS	

Among the overlapping pathways, the most disease-relevant pathways were those related to MHC classes I and II protein complexes, protein antigen binding and receptor activity. Where the personalised approach identified the relevance of these pathways in all three analyses, the combined method identified them as significant only in the TC analysis. Moreover, the combined method failed to identify the overarching pathway, ‘antigen processing and presentation’, as significant, which was found to be significant in all three analyses using the personalised approach. In addition, other interesting and relevant pathways that were identified by the personalised approach were not found using the combined method in any of the analyses.

In particular, the combined method was also unable to identify one of the most basic pathways related to immunological diseases, ‘immune response’, or any of its related pathways in any of the analyses. In fact, the ‘Type 1 diabetes’ pathway was also not found as significant in any of the analyses using the combined method. On the contrary, the personalised approach found the ‘immune response’ pathway as highly significant in all three analyses and many related pathways in at least one analysis. It also identified the T1D pathway as highly enriched in all three analyses.

### Enriched pathways identified by the personalised approach

**Fig. 3:**
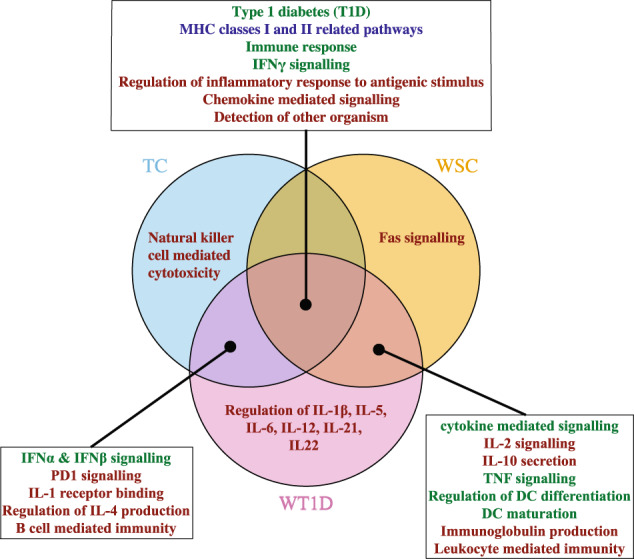
Venn diagram illustrating disease-relevant pathways specific to a certain analysis or overlapping between analyses using the personalised approach. Here, pathways in blue text refer to those found enriched by the personalised approach, combined method as well as Kallionpää et al.^31^; green text refer to those found enriched by the personalised approach and Kallionpää et al.^31^; and red text refer to those found enriched by only the personalised approach. Full lists of enriched pathways are found in Supplementary Data.

While analysing *Datasets 1* and *2*, several disease-relevant and intriguing pathways were identified as enriched using the personalised approach in either all three analyses, only two analyses or uniquely in one analysis. In order to establish relevance of these results, they were cross-validated with the results from Kallionpää et al.^31^. The differentially expressed lists of genes from the analyses in the article corresponding to our TC, WSC and WT1D analyses were subjected to a Fisher’s exact test using the pathways from the MSigDB^25,38^ to ensure comparability. Their gene-level results identified 386, 432, and 824 pathways as enriched in the TC, WSC, and WT1D analyses, respectively (Table 1). These pathways overlapped significantly (*p*-values < 0.0001 using Fisher’s exact test) with the enriched pathways found by the personalised approach in all analyses, as well as the enriched pathways identified in the TC analysis using the combined method (*p*-value < 0.01), but overlapped insignificantly with the results from time-window analyses using the combined method (Table 2). Essentially, Kallionpää et al.^31^ were able to identify many of the significant pathways identified using the personalised approach. However, they mostly identified only the overarching pathways, but not the related pathways with more specialised functions. In some cases, they identified the significance of certain pathways in different analyses than the personalised approach. For instance, the T1D, as well as MHC classes I and II related pathways were found enriched (FDR < 0.05) in only the WT1D analysis, whereas our method found it in all three analyses. The interesting pathways discussed below that were identified using the personalised approach is illustrated in Fig. 3 and those identified by Kallionpää et al.^31^ and the combined method are highlighted with different colours.

The personalised approach identified significant (FDR < 0.05) pathways related to immune response, interferon-*γ* (IFN*γ*) signalling, regulation of inflammatory process to antigenic stimulus, chemokine mediated signalling, and detection of other organism, in all three analyses suggesting their relevance at all stages of the disease (see Supplementary Data). Of these, Kallionpää et al.^31^ only identified immune response and IFN*γ* signalling related pathways as enriched (FDR < 0.05) in all analyses and detection of other organism pathway was found enriched (FDR < 0.05) in only the WT1D analysis.

Multiple interesting overarching pathways were identified as enriched by the personalised approach uniquely in the time-windows right before seroconversion and T1D diagnosis, which were also found by Kallionpää et al.^31^ in at least one of the analyses. These include the pathways related to cytokine mediated signalling, TNF signalling, regulation of dendritic cell (DC) differentiation, and DC maturation. However, in contrast to Kallionpää et al.^31^ results, the personalised approach was also able to highlight specific cytokine pathways that could be involved in the cytokine mediated signalling, as well as possible pathways necessary to regulate/conduct the immune response. In particular, IL-2 and IL-10 related pathways were enriched along with immunoglobulin production, and leucocyte mediated immunity.

Intriguingly, the personalised methods found several pathways that were uniquely enriched during the early prognosis of T1D and in the 6 months window before T1D diagnosis. While IFN*γ* signalling was found significant at all stages of the disease, interferon-*α* (IFN*α*) and interferon-*β* (IFN*β*) signalling were enriched only in the TC and WT1D analyses using the personalised approach, whereas Kallionpää et al.^31^ associate their relevance at all stages. In addition, we found other T1D-associated pathways, such as PD1 signalling, IL-1 receptor binding, regulation of IL-4 production and positive regulation of B cell mediated immunity, to be enriched in the TC and WT1D analyses. However, Kallionpää et al.^31^ were unable to detect them.

Furthermore, distinct disease-relevant pathways were determined as uniquely enriched before seroconversion, before T1D diagnosis or during the early stages of T1D progression using only the personalised approach. Specifically, pathways related to natural killer cell-mediated cytotoxicity and Fas signalling were found to be uniquely significant during the early stages of T1D progression and before seroconversion, respectively. Most strikingly, pathways regulating the production of multiple different pro-inflammatory and anti-inflammatory cytokines, such as Interleukin-1, -1*β*, -2, -4, -5, -6, -10, -12, -21, -22, as well as the related overarching pathways, were found enriched in the 6 months before clinical onset of T1D, where more than half of the cytokine pathways were unique to this time-window.

For assessing the generalisability of the results from our personalised approach, we performed TC analysis also on third independent dataset, namely *Dataset 3*, and performed Spearman’s rank correlation test between the FDR values of all pathways obtained from analysing *Datasets 1* and *3*. The Spearman’s rank correlation value (*ρ*) for all pathways was 0.203, which was found to be highly statistically significant with *p*-value < 10^−15^. The same correlation test performed on the 32 disease-relevant pathways (highlighted in Supplementary Data) found enriched in TC analysis using *Dataset 1* resulted in *ρ* = 0.604, which was also found highly statistically significant with *p*-value < 10^−3^. Most importantly, many of the disease-relevant pathways found enriched in the TC analysis using *Dataset 1* were found enriched using *Dataset 3* as well; including the T1D pathway; and pathways related to immune response; interferon-*α*, -*β* and -*γ* signalling; antigen processing and presentation; cytokine-mediated signalling; and IL-1 and IL-4 production.

### Type 1 diabetes pathway

**Fig. 4:**
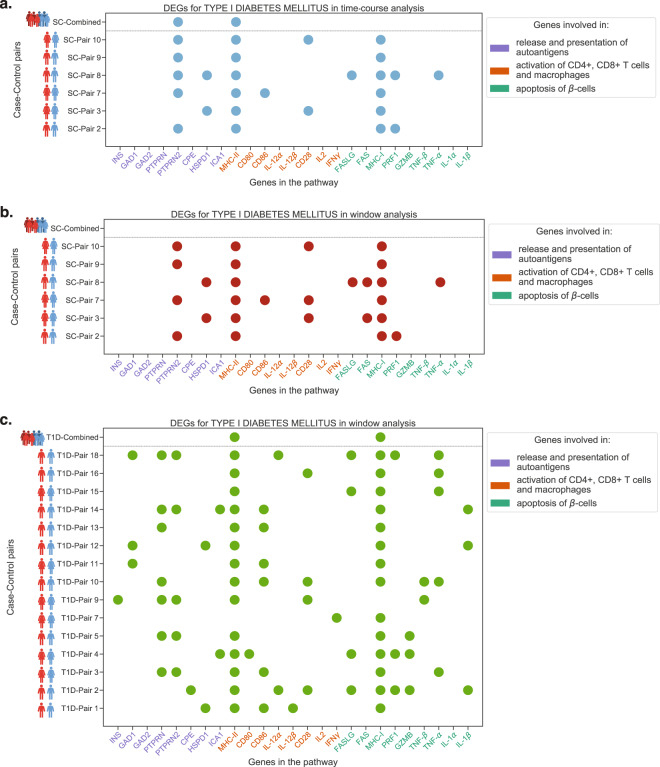
A comparative visualisation of the DEGs between the two approaches for the T1D pathway in all three analyses. **a** DEGs from T1D pathway in the TC analysis using *Dataset 1*, (**b**) DEGs from T1D pathway in the WSC analysis using *Dataset 1* and (**c**) DEGs from T1D pathway in the WT1D analysis using *Dataset 2*. A coloured dot signifies that the gene is DE in the corresponding case-control pair. Here, the HLA genes from MHC classes I and II are not marked individually, but grouped into their two major classes for convenience; and a class is shown as DE in a case-control pair when at least one probe-set from any HLA gene of the class was found DE in that pair. Also, pairs from *Dataset 1* and *Dataset 2* are prefixed with ‘SC-’ and ‘T1D-’, respectively.

The type 1 diabetes pathway was found enriched in all three analyses of *Datasets 1* and *2*, as well as TC analysis of *Dataset 3* using the personalised approach. However, the combined method did not find it significant in any of the analyses and Kallionpää et al.^31^ found its significance only in the late stages of the disease, i.e., window before clinical onset of T1D. Fig. 4 shows the genes that were identified as differentially expressed (BF-score > 4) in each analysis per case-control pair (coloured dots) of *Datasets 1* and *2*. These figures clearly illustrate that only a small fraction of the pathway’s genes are differentially expressed (DE) in most of the case-control pairs and only a subset of these genes are DE in each child. Moreover, the subset of DE genes varies from one pair to another. It is not clearly understood how the presence of certain genes influence that of the other genes, therefore it is not easy to predict which genes in a pathway are selectively or necessarily expressed. When the T1D pathway genes were functionally divided into 3 main sub-processes: release and presentation of autoantigens; activation of CD4+, CD8+ T cells and macrophages; and apoptosis of *β*-cells, it was noticed that at least one gene from each sub-process was identified as DE in each pair. Some pairs did not differentially express any of the (auto)antigen encoding genes, which could indicate an environmental source of (auto)antigens instead of genetic. Similar phenomena may be expected from most other pathways. As an additional example, IFN*γ* signalling pathway has been depicted in Supplementary Figs. 3 and 4.

The combined method identified only those genes as DE that were DE in almost all the pairs (Fig. 4). Therefore, for a pathway to be recognised as enriched using the combined method, a significant number of the genes in the pathway would need to be DE in most of the pairs, which may not be how heterogeneous diseases, such as T1D, affect pathways.

## Discussion

The results of this paper demonstrate that a personalised approach of identifying differentially expressed genes (DEGs) and summarising them on a pathway-level can reveal more insight into the progression of heterogeneous diseases, such as type 1 diabetes (T1D), than commonly used non-personalised approaches that assume differences between cases and controls to be consistent across the whole study population, such as the combined method presented in this paper. Even though a significant number of pathways identified by the two approaches overlapped, the combined method was unable to identify the significance of most of the disease-relevant and interesting pathways that were identified by the personalised approach in all the analyses. The combined model identified DEGs in a strict manner that may also be biologically unrealistic, which probably impeded its ability to pinpoint most of the disease-relevant and intriguing pathways.

For validation, the results from the personalised approach (*Datasets 1* and *2*) were compared to that of the results from Kallionpää et al.^31^, who analysed the same datasets using a rank product algorithm introduced by Breitling et al.^43^ for identifying DEGs, which cannot account for neither the dynamics of the time-course data nor the heterogeneity. Moreover, they estimated unobserved values in time-window analyses via linear inter-/extrapolation, where we applied Gaussian process modelling, which is known to be more robust. Significant number of pathways identified as enriched by the personalised approach overlapped with the Kallionpää et al.^31^ results. However, while Kallionpää et al.^31^ identified mostly the overarching pathways as enriched, the personalised approach recognised significance of the overarching pathways, as well as more specialised pathways that illustrate the intrinsic mechanisms by which the disease develops. Also, the analysis of *Dataset 3* using our personalised approach demonstrated the generalisability of our pathway-level results concerning other T1D datasets.

Below, we discuss some of the interesting pathways found enriched by the personalised approach and explore their relevance in terms of T1D, as well as the stages of the disease they were found enriched in.

Considering that T1D is a complex autoimmune disease characterised by insulitis, the chronic inflammation of the pancreatic islets of Langerhans caused by autoreactive CD4+ and CD8+ T cells^35,36,44,45^, pathways related to immune response are expected to be enriched along with the T1D pathway. While these particular pathways were not found enriched using the combined model, it did identify interesting and relevant pathways in the TC analysis that largely fall under, but not include, the overarching ‘antigen processing and presentation’ pathway. These were the pathways involving MHC class protein and dendritic cell (DC) maturation. Even though these pathways are highly relevant in the context of the disease, they mostly represent only the initiating events in the development of the disease: release of autoantigens; their uptake by antigen presenting cells (APCs), such as DCs, for antigen presentation in a complex with MHC class proteins^44^; and migration of DCs to pancreatic lymph nodes (pLN) to activate *β*-cell specific autoreactive T cells^35,44^, known as DC maturation^46^. Meanwhile, other important and disease-relevant pathways are underrepresented using the combined model.

The personalised approach also finds the above-mentioned pathways enriched in its analyses, including immune response related and T1D pathways, along with many other disease-relevant pathways. In all the analyses, our approach identifies the pathways related to IFN*γ* signalling and chemokine-mediated signalling as enriched. IFN*γ* is produced by autoreactive CD4+ and CD8+ T cells^47^ and is believed to play a key role in driving the autoimmune pathogenesis of T1D^35,44,45,47–50^, even though it is not considered solely a pro-inflammatory cytokine^47^. IFN*γ* also results in local upregulation of chemotactic cues that induce immune cell migration to the islets, for instance via chemokine mediated signalling, where *β*-cells produce certain chemokines that can accelerate or block T1D progression^35^. Fascinatingly, our approach also identified a pathway, ‘detection of other organism’, which connotes an existing postulation that environmental factors, such as microbial infections, can trigger the disease process leading to T1D in genetically susceptible individuals^35,44,51^.

One of the most interesting questions that are asked in T1D studies is regarding the changes that transpire in the time-window leading up to life-changing events, such as seroconversion and clinical onset of T1D. Using the personalised approach, multiple immunologically relevant pathways were revealed to be uniquely enriched in both the time-windows of interest, such as TNF signalling, where TNF-*α* has been linked to the development of T1D^35,44,48,50,52^; DC differentiation and maturation^46,48^; and cytokine-mediated signalling^35,44,45^, which acts like an all-encompassing, but vague, pathway for all cytokines. The method was able to determine additional relevant pathways in these two time-windows that were not identifiable by Kallionpää et al.^31^ results: immunoglobulin production, as well as IL-2 and IL-10 regulating pathways. In fact, it is the increase in production of islet autoantibodies or immunoglobulin that marks the seroconversion event in the life of an individual susceptible to T1D^31^. Meanwhile, enrichment of IL-2 and IL-10 signalling pathways before seroconversion indicates the possible anti-inflammatory processes that occur to resist the progression of the disease. IL-10 is an anti-inflammatory cytokine secreted primarily by Tregs and *β*-cell autoantigen recognising CD4+ T cells^45^. It inhibits the production of multiple pro-inflammatory cytokines, including IFN*γ*, TNF-*α*, IL-5, IL-1*β*, etc.^50^, and is only marginally less prevalent in T1D patients studied at the time of diagnosis than in healthy subjects^45^. IL-2 is a cytokine that can lead to prevention or pathogenesis of the disease depending on its own concentration, the concentrations of other local cytokines^53–55^ and polymorphisms in the genes of its pathway^45^. In low dose, IL-2 signalling is believed to rescue insulin secretion^54,55^. However, it may result in accelerated autoimmune tissue destruction in the time-window before diagnosis due to the enriched regulation of IL-1 signalling in that time-window as it enhances IL-2 production^50,54^.

Our results identify increased number of pathways enriched in the window before T1D onset as compared to the window before seroconversion, demonstrating the mayhem that precedes a clinical diagnosis. Especially, the number of cytokine regulating pathways were increased manifold, where more than half were unique to this time-window. Along with anti-inflammatory cytokines, such as IL-10 and IL-4^48,50,56^, many pro-inflammatory cytokine regulating pathways were enriched, such as IL-1, IL-1*β*, IL-5^50^, IL-6^48,50^, IL-12^46^, IL-21^35,44,57^, IL-22^50^, IFN*γ*, TNF-*α*. In the absence of IFN*γ* and TNF-*α*, cytokines IL-2, IL-1*β* and IL-6 are considered anti-inflammatory^35,44,48,50,54,55^, but in their presence, these cytokines aggravate the inflammatory disease pattern, which is probably the case in the time-window before T1D diagnosis.

Some of the pathways that were found enriched in the time-window before T1D diagnosis were also found enriched during the early stages of T1D progression using the personalised approach, possibly indicating that key players from late stages of the disease may already be detected at the early stages. These included both pro-inflammatory and anti-inflammatory pathways, such as those of IL-1 and IL-4, as well as IFN*α* and PD-1 signalling. IL-1 is a pro-inflammatory cytokine that enhances the production of IL-2, encourages B cell proliferation, and increases immunoglobulin production^50,54^; whereas IL-4 is an anti-inflammatory Th2 cytokine that inhibits autoimmunity by downregulating the production of pro-inflammatory cytokines, such as IL-1, IL-6, and TNF-*α*^48,50,56^. Through mice studies, IFN*α* and PD-1 signalling pathways have been established as important contributors to T1D pathogenesis from an early stage of the disease^46,58–60^. Where upregulation of IFN*α* in pLN is an initiator of the pathogenesis^59^, upregulation of programmed cell death protein 1 (PD-1) signalling prevents T1D and promotes self-tolerance by suppressing the expansion and infiltration of autoreactive T cells in the pancreas^46,60,61^. In fact, blocking IFN*α* signalling before clinical T1D onset has been shown to prevent *β*-cell apoptosis or even abort T1D progression^58^. In addition, PD-1 pathway has been proposed as a target for a new therapy for preventing and modulating autoimmunity^61^.

Fascinatingly, natural killer (NK) cell mediated cytotoxicity pathway was found to be uniquely enriched during the early stages of T1D. NK cells are believed to be involved in multiple steps of the immune-mediated attack causing T1D as they are known to interact with antigen-presenting T cells, secrete pro-inflammatory cytokines and induce apoptosis in the target cells^62,63^. Similarly, Fas signalling pathway was found to be uniquely enriched before seroconversion. Since it is one of the pathways mediated by autoreactive CD8+ T cells that is directly involved in the destruction of *β*-cells^35,44,46^, it demonstrates that *β*-cell killing can be observed much before the clinical onset of T1D.

Even though the personalised approach is able to identify many immunologically-relevant and disease-relevant pathways, it has scope for further development. The current implementation assumes Gaussian distributed data; it may be possible to improve the accuracy of differential gene expression detection for datasets that have notably non-Gaussian characteristics either by using a different likelihood model or by performing appropriate transformations. In addition, the proposed approach has been implemented for a matched case-control setting. However, with small modifications to the model, it could be extended to a non-matched case-control setting, where each case is compared to all the controls in the dataset.

## Methods

### Gaussian process regression

A Gaussian process is a generalisation of the Gaussian distribution. It can be seen as defining a distribution over functions and inference taking place directly in the space of functions^64^. We denote ${\bf{X}}={\left({x}_{{t}_{1}},{x}_{{t}_{2}},\ldots ,{x}_{{t}_{N}}\right)}^{{\rm{T}}}\in {{\mathbb{R}}}^{N}$ as a vector of noisy measurements for a particular probe-set, which were measured at *N* time points, **T** = (*t*_1_, *t*_2_, …, *t*_*N*_). The GP is defined as

1 $$f(t) \sim GP(\mu (t),k(t,t^{\prime} )),$$

which represents a distribution over function samples *f*(**T**) = (*f*(*t*_1_), *f*(*t*_2_), …, *f*(*t*_*N*_)). Here, *μ*(*t*) is the mean which we assume as zero and $k(t,t^{\prime} )$ is a positive semi-definite kernel function, which has kernel parameters ***θ***, i.e., $k(t,t^{\prime} | {\boldsymbol{\theta }})$. We assume additive Gaussian observation noise *ϵ*, where Gaussian observation is defined as

2 $$x=f(t)+\epsilon ,$$

where $\epsilon \sim {\mathcal{N}}(0,{\sigma }_{\epsilon }^{2})$. Gaussian process regression modelling involves placing a Gaussian prior, $f({\bf{T}}) \sim {\mathcal{N}}(0,{{\bf{K}}}_{{\bf{T}},{\bf{T}}}({\boldsymbol{\theta }}))$ over the true model, where the elements of the covariance matrix are defined by the kernel ${[{{\bf{K}}}_{{\bf{T}},{\bf{T}}}({\boldsymbol{\theta }})]}_{i,j}=k({t}_{i},{t}_{j}| {\boldsymbol{\theta }})$. Here, we use the popular squared exponential kernel, which is defined as

3 $$k({t}_{i},{t}_{j}| {\boldsymbol{\theta }})={\sigma }_{{\rm{se}}}^{2}\exp \left(-\frac{{({t}_{i}-{t}_{j})}^{2}}{2{\ell }_{{\rm{se}}}^{2}}\right),$$

where *ℓ*_se_ is the length-scale parameter that controls the smoothness and ${\sigma }_{{\rm{se}}}^{2}$ is the signal variance of the kernel. Hence, the kernel parameters are ${\boldsymbol{\theta }}=({\ell }_{{\rm{se}}},{\sigma }_{{\rm{se}}}^{2})$.

Given the observed data **X**, the measurement time points **T** and test time points T_*_, we obtain the posterior distribution $f({{\bf{T}}}_{* })| {\bf{X}} \sim {\mathcal{N}}({{\boldsymbol{\mu }}}_{* },{{\boldsymbol{\Sigma }}}_{* })$ defined by

$${{\boldsymbol{\mu }}}_{* }={{\bf{K}}}_{{{\bf{T}}}_{* },{\bf{T}}}{({{\bf{K}}}_{{\bf{T}},{\bf{T}}}+{\sigma }_{\epsilon }^{2}{\bf{I}})}^{-1}{\bf{X}}$$

4 $${{\boldsymbol{\Sigma }}}_{* }={{\bf{K}}}_{{{\bf{T}}}_{* },{{\bf{T}}}_{* }}-{{\bf{K}}}_{{{\bf{T}}}_{* },{\bf{T}}}{({{\bf{K}}}_{{\bf{T}},{\bf{T}}}+{\sigma }_{\epsilon }^{2}{\bf{I}})}^{-1}{{\bf{K}}}_{{\bf{T}},{{\bf{T}}}_{* }},$$

where we denote **K**_**T**,**T**_ = **K**_**T**,**T**_(***θ***) for brevity and ${{\bf{K}}}_{{{\bf{T}}}_{* },{\bf{T}}}={{{\bf{K}}}_{{\bf{T}},{{\bf{T}}}_{* }}}^{{\rm{T}}}$ encodes the cross-correlations between measured and test time points.

### Prior specification

The gene expression data is first centred to zero by subtracting the mean of the data for GP fitting. This is done independently for the case, control and pooled (case and control) data. For the length-scale (*ℓ*_se_) parameter of the squared exponential kernel, we specify a Gaussian prior (*μ* = 30, *σ*^2^ = 6). We chose the value of *μ* to correspond to 30 weeks which results in a small probability of short length-scales and provides a reasonable range of feasible length-scales. The magnitude (${\sigma }_{{\rm{se}}}^{2}$) parameter is assigned a square root student-*t* prior (*μ* = 0, *σ*^2^ = 1, and *ν* = 20). The noise variance parameter is assigned a scaled inverse chi-square prior (*σ*^2^ = 0.01 and *ν* = 1) to restrict it to smaller magnitudes. We use the same (hyper)parameter priors for the case, control, as well as joint GPs.

### Marginal likelihood estimation using central composite design

The choice of hyperparameters has a significant effect on the resulting kernel with respect to smoothness and magnitude of the kernel. Computing the exact marginal likelihood (ML) is computationally intractable due to the marginalisation over the hyperparameters. Another approach to solving this problem, would be to simply maximise the ML with respect to the hyperparameters. Such an approximation is known as type II maximum likelihood (ML-II) and can lead to overfitting^64^ as it may underestimate/ignore uncertainty of hyperparameters, especially for small sample sizes common in biomedical studies. In fact, in our analysis, the ML-II approach failed to generate satisfactory estimates in many instances and hence was unsuitable for our purposes. To overcome this problem and to ensure appropriate modelling of kernel hyperparameters, we make use of a form of numerical integration approximation, called central composite design (CCD), for posterior prediction as proposed in Rue et al.^65^ and Vanhatalo et al.^66^, to approximate the exact marginal likelihood. CCD assumes a split-Gaussian posterior for log-transformed hyperparameters and defines a set of *R* points ${\{{{\boldsymbol{\theta }}}_{r}\}}_{r = 1}^{R}$ (fractional factorial design, the mode, and so-called star points along whitened axes) that allow for estimating the curvature of the posterior distribution around its mode (see refs. ^65,66^). We estimate the ML by using the *R* CCD points that are located around the high-probability region of the posterior (which is the integrand in the ML integral) but by replacing the split-Gaussian approximation used for posterior predictions with the exact product of likelihood and prior. In other words, we take the weighted sum of the posterior probability evaluated at the *R* points of the hyperparameter, which are weighted by the integration weights. For a model *M* with data **X**, the estimated ML is given by

5 $$p({\bf{X}}| M)=\int{p}({\bf{X}}| M,{\boldsymbol{\theta }})p({\boldsymbol{\theta }}| M)d{\boldsymbol{\theta }}\,\approx \,{\Sigma }_{r = 1}^{R}p({\bf{X}}| M,{{\boldsymbol{\theta }}}_{r})p({{\boldsymbol{\theta }}}_{r}| M){\Delta }_{r},$$

where $p({\bf{X}}| M,{{\boldsymbol{\theta }}}_{r})={\mathcal{N}}(0,{{\bf{K}}}_{{\bf{T}},{\bf{T}}}({{\boldsymbol{\theta }}}_{r})+{\sigma }_{\epsilon }^{2}{\bf{I}})$ and *Δ*_*r*_ is *r*th integration weight that corresponds to the volume of hyperparameter space allocated to the *r*th point. The obtained estimated ML for each model is then used to compute a Bayes factor score, which is used for model selection and for identifying differentially expressed genes (DEGs) as discussed below.

### Personalised approach to identifying DEGs in time-course analysis using ML ratio

To identify if a feature (i.e., probe-set or gene) is differentially expressed (DE) between a matched case-control pair, we fit a *joint* and *separate* model to the expression data and identify which model better explains the observed data. The *joint* model involves fitting a Gaussian process over all the data points (i.e., case and control data), whereas the *separate* model involves independently fitting a GP to only the data points corresponding to the cases and fitting another GP to only the data points corresponding to the control. After the fitting, model selection is performed to choose between the *joint* and *separate* model. If the *joint* model is chosen, we conclude that the case and control expressions for the specific feature comes from the same process and hence is not differentially expressed. Alternatively, if the *separate* model is chosen, we conclude that the case and control expressions for the corresponding feature comes from different processes and hence is differentially expressed. Assume two independent models, *M*^*A*^ and *M*^*B*^, which are fit to the case and control time-course of a particular feature, **x**^**A**^ and **x**^**B**^, respectively. Also, let a *joint* model, *M*^*S*^, be fit to the pooled data **x**^**S**^ = (**x**^**A**^; **x**^**B**^). A standard statistical test would compare models *M*^*A*^ and *M*^*B*^ (*separate* models) against the *joint* model, *M*^*S*^. Hence, the null hypothesis would correspond to no differential expression and the alternate hypothesis would correspond to the presence of differential expression^19^.

To perform model selection, we compute a Bayes factor score for each feature and case-control pair separately. This is calculated as the log ratio of the marginal likelihoods of the *separate* and *joint* models,

6 $${\mathrm{BF}}\hbox{-}{\mathrm{score}}\,=\,{\mathrm{log}}\,\frac{p({\bf{x^A}}| M^A)p({\bf{x^B}}| M^B)}{p({\bf{x^S}}| M^S)}.$$

This gives us a score for quantifying the differential expression of each feature. We use a threshold of 4 (≈54.598 in the linear scale) to identify DE features, which corresponds to strong evidence for rejecting the null hypothesis as stated in Kass et al.^67^.

In case of probe-set data, we now map the probe-sets to their corresponding gene names. If multiple probe-sets map to the same gene name, we choose the probe-set with largest BF-score to represent the gene. This is done independently for each case-control pair, which allows the flexibility of choosing different probe-sets between pairs to represent the same gene.

### Personalised approach to identifying DEGs in time-window analyses using KL divergence

In addition to the TC analysis, we also detect disrupted pathways within certain time-windows. This approach could potentially be used to identify the pathways that are affected before a significant event in the prognosis of a disease (e.g., seroconversion and clinical onset of T1D) and hence, can have applications in predictive medicine. The size of the time-window can be chosen as any appropriate duration. Here, we chose to detect significant genes by comparing the expression levels of features between each case-control pair in a 26 week (approx. 6 months) time-window prior to the seroconversion event and clinical disease onset. We compute the posterior mean and variance of the latent variables of the Gaussian processes within the chosen time-window, as described in Eq. (4), using the representative points of the hyperparameters. We then compute the weighted sum for the predictive mean and variance weighted on the approximative posterior and the integration weights, and approximate the posterior distribution as

7 $$\begin{array}{l}{p}{({f}{({\bf{T}}_*)}{|{\bf{X}},M)}} \approx {\mathcal{N}}{({f}({{\bf{T}}_*)|}}{\boldsymbol{\mu}}_{\text{pred}}, {\mathbf{\Sigma}}_{\text{pred}}),\\{{\boldsymbol{\mu}}_{\text{pred}} = {\sum \limits_{r=1}^R}{\mu_{*}^{r}{q}}(\theta_{r} | M )\Delta_r}\\{{\mathbf{\Sigma}}_{\text{pred}} = {\sum \limits_{r=1}^R}{\Sigma_{*}^{r}{q}}(\theta_{r} | M )\Delta_r}\end{array}$$

where ${{\mu}_\ast^{r}}$ and ${\Sigma}_\ast^{r}$ are calculated, as in Eq. (4), evaluated with hyperparameter value ***θ***_*r*_; *q*(***θ***_*r*_∣*M*) is the split-Gaussian approximative posterior; and T_*_ defines a time discretisation for the 26 week time interval (we use 26 time points, i.e., a resolution of one week). Comparisons for the time-window predictions between *separate* (comprising of separate GP fittings for the cases and controls) and *joint* (single GP fitting the pooled case and control data points) models can be made by comparing the distributions using the Kullback–Leibler (KL) divergence^68^. The Kullback–Leibler divergence for any two distributions, ${\mathcal{P}}$ and ${\mathcal{Q}}$, can be defined as

8 $${\mathcal{D}}_{KL}({\mathcal{P}}|| {\mathcal{Q}})={\int \nolimits_{-\infty }^{\infty}} p(x){\log}\,\frac {p(x)}{q(x)}{d}x,$$

where *p* and *q* are the corresponding densities. To examine the expression level of a probe-set in the time-window, we compare the predictive distributions (Eq. (7)) for the *joint* model against the *separate* model in the time-window, by calculating a continuous score obtained using the symmetric KL divergence,

9 $$\frac{1}{2}{{\mathcal{D}}}_{{KL}}({\mathcal{P}}| | {\mathcal{Q}})+\frac{1}{2}{{\mathcal{D}}}_{{KL}}({\mathcal{Q}}| | {\mathcal{P}}).$$

Therefore, to compute the symmetric KL divergence between the *separate* and *joint* model, we assume two multivariate normal distributions: one for the *separate* model, represented by ${{\mathcal{M}}}_{0}$ (previously denoted by *M*^*A*^ and *M*^*B*^); and one for the *joint* model, represented by ${{\mathcal{M}}}_{1}$ (previously denoted by *M*^*S*^) with dimension equal to twice the number of weeks in the time-window. In ${{\mathcal{M}}}_{0}$, let ${{\boldsymbol{\mu }}}_{* }^{{\bf{case}}}$ and ${{\boldsymbol{\Sigma }}}_{* }^{{\bf{case}}}$ be the predictive mean and covariance matrix (from Eq. (7)) for the case GP with the test points taken weekly from the first to the last week of the combined data points. Similarly, for the control GP (of ${{\mathcal{M}}}_{0}$) and joint GP (of ${{\mathcal{M}}}_{1}$), we have ${{\boldsymbol{\mu }}}_{* }^{{\bf{control}}}$ and ${{\boldsymbol{\Sigma }}}_{* }^{{\bf{control}}}$, as well as ${{\boldsymbol{\mu }}}_{* }^{{\bf{joint}}}$ and ${{\boldsymbol{\Sigma }}}_{* }^{{\bf{joint}}}$, respectively. The predictive distribution of ${{\mathcal{M}}}_{0}$ can be written as

10 $${{\mathcal{M}}}_{0}={\mathcal{N}}\left({{\boldsymbol{\mu }}}_{* }^{{\boldsymbol{0}}}=\left[\begin{array}{c}{{\boldsymbol{\mu }}}_{* }^{{\bf{case}}}\\ {{\boldsymbol{\mu }}}_{* }^{{\bf{control}}}\end{array}\right],{{\boldsymbol{\Sigma }}}_{* }^{{\boldsymbol{0}}}=\left[\begin{array}{cc}{{\boldsymbol{\Sigma }}}_{* }^{{\bf{case}}}&{\boldsymbol{0}}\\ {\boldsymbol{0}}&{{\boldsymbol{\Sigma }}}_{* }^{{\bf{control}}}\end{array}\right]\right).$$

Similarly, the predictive distribution of ${{\mathcal{M}}}_{1}$ can be written as

11 $${{\mathcal{M}}}_{1}={\mathcal{N}}\left({{\boldsymbol{\mu }}}_{* }^{{\bf{1}}}=\left[\begin{array}{c}{{\boldsymbol{\mu }}}_{* }^{{\bf{joint}}}\\ {{\boldsymbol{\mu }}}_{* }^{{\bf{joint}}}\end{array}\right],{{\boldsymbol{\Sigma }}}_{* }^{{\bf{1}}}=\left[\begin{array}{cc}{{\boldsymbol{\Sigma }}}_{* }^{{\bf{joint}}}&{\boldsymbol{0}}\\ {\boldsymbol{0}}&{{\boldsymbol{\Sigma }}}_{* }^{{\bf{joint}}}\end{array}\right]\right).$$

The Kullback-Leibler divergence for any two multivariate normal distributions, say ${{\mathcal{M}}}_{0}$ and ${{\mathcal{M}}}_{1}$, can be computed directly from the formula^69^

12 $${{\mathcal{D}}}_{KL}\left({{\mathcal{M}}}_{0}| | {{\mathcal{M}}}_{1}\right)=\frac{1}{2}\left({\rm{tr}}({{{\boldsymbol{\Sigma }}}_{* }^{{\bf{1}}}}^{-1}{{\boldsymbol{\Sigma }}}_{* }^{{\boldsymbol{0}}})+{({{\boldsymbol{\mu }}}_{* }^{{\bf{1}}}-{{\boldsymbol{\mu }}}_{* }^{{\boldsymbol{0}}})}^{T}{{\boldsymbol{\Sigma }}}_{* }^{{{\bf{1}}}^{-{\bf{1}}}}({{\boldsymbol{\mu }}}_{* }^{{\bf{1}}}-{{\boldsymbol{\mu }}}_{{\boldsymbol{0}}}^{{\bf{control}}})-k+\mathrm{ln}\,\left(\frac{\det \mathop{{\boldsymbol{\Sigma }}}\nolimits_{* }^{{\boldsymbol{1}}}}{\det \mathop{{\boldsymbol{\Sigma }}}\nolimits_{* }^{{\boldsymbol{0}}}}\right)\right),$$

where ${{\boldsymbol{\mu }}}_{* }^{{\boldsymbol{0}}}$ and ${{\boldsymbol{\Sigma }}}_{* }^{{\boldsymbol{0}}}$ are the parameters of ${{\mathcal{M}}}_{0}$, and ${{\boldsymbol{\mu }}}_{* }^{{\bf{control}}}$ and ${{\boldsymbol{\Sigma }}}_{* }^{{\bf{1}}}$ are the parameters of ${{\mathcal{M}}}_{1}$. Also, *k* is the dimension of the multivariate Gaussian, which in our case is 2 × 26 (weeks); ${\rm{tr}}(\cdot )$ and $\det$ refer to the trace and determinant of the matrix, respectively.

The symmetric KL divergence gives a KL-score for each feature. The KL-score can be written as:

13 $${\mathrm{KL}}\hbox{-}{\mathrm{score}}\,=\,\frac{1}{2}\left({{\mathcal{D}}}_{{KL}}\left({{\mathcal{M}}}_{0}| | {{\mathcal{M}}}_{1}\right)+{{\mathcal{D}}}_{{KL}}\left({{\mathcal{M}}}_{1}| | {{\mathcal{M}}}_{0}\right)\right).$$

KL-scores do not have a similar interpretation as the Bayes factor. Therefore, we empirically set a threshold to identify differential expression prior to an event by taking the mode of the KL-scores of the features (from all case-control pairs) that have a BF-score in the range of +/− 1 of the chosen BF-score threshold (in our case, BF-scores in the range 3 to 5 as the threshold is set to 4). The objective of this is to find an appropriate KL-score threshold from the features that are borderline DE (or not) according to the BF-scores computed in the TC analysis. Note, however, that a specific value for the threshold is not critical as the pathway-level enrichment analysis automatically balances liberal or stringent threshold values.

In case of probe-set data, we now map the DE probe-sets to their corresponding gene names. However, in the case of multiple probe-sets mapping to the same gene name, we choose the probe-set with the largest KL-score to represent the gene.

### Pathway analysis for personalised differential gene expression results

We propose an empirical hypothesis testing method that can identify statistically enriched pathways from DE genes (DEGs) that are identified for all case-control pairs separately. We define an overall enrichment score for each pathway using the DEGs from each case-control pair and a statistic we call *adjusted geometric mean*. Our enrichment analysis uses the number of DEGs from each case-control pair that overlap a given pathway. To account for the fact that a higher number of DEGs in a case-control pair leads to a higher probability of overlap with a pathway, we divide the raw number of DEGs from a case-control pair in a pathway by the total number of DEGs in that case-control pair. Thus, we compute the *scaled pathway overlap* *f*_*i*,*j*_ for the *j*^th^ case-control pair and *i*^th^ pathway as

14 $${f}_{i,j}=\frac{{{\rm{overlap}}}_{i,j}}{{\rm{diff}}.\,{\rm{exp}}.\,{\rm{genes}}_{j}}+\alpha ,$$

where overlap_*i*,*j*_ refers to the number of DEGs in the *j*th case-control pair belonging to the *i*th pathway, diff.exp.genes_*j*_ refers to the number of DEGs in the *j*th case-control pair (assumed to be larger than 0 for all *j*), and *α* is a small constant (*α* = 10^−6^ in our analyses). Assuming *m* case-control pairs, we define *adjusted geometric mean* of the *i*th pathway as

15 $${{\rm{adj.geo.mean}}}_{i}={\left(\mathop{\prod }\limits_{j = 1}^{m}{f}_{i,j}\right)}^{\frac{1}{m}}.$$

The *adjusted geometric mean* ensures that no case-control pair dominates the overall enrichment score and helps to take into account the different number of DEGs from each case-control pair.

After the *adjusted geometric mean* scores for each pathway are computed, we identify the statistically enriched pathways by performing a permutation test and obtain p-values for each pathway. Let ${\bf{S}}\in {{\mathbb{R}}}^{G\times m}$ denote a matrix that stores the BF-scores or KL-scores from Eqs. (6) and (13) (where *G* corresponds to the total number of features and *m* is the number of case-control pairs) such that **S**_*g*,*j*_ contains the BF-scores or KL-scores for the *g*th feature and the *j*th case-control pair. Our permutation strategy reorders the feature labels of the rows, which retains the possible correlations among the scores for the features across the case-control pairs. In other words, we fix the matrix **S** and shuffle just the associated features such that each row is randomly assigned a feature. In case of probe-set data, after the reordering (shuffling), the probe-sets are again assigned to gene names and the enrichment scores (*adjusted geometric mean* scores) are computed. This process of feature label shuffling and computing enrichment scores is repeated 100,000 times to get the permutation distribution that is used to compute the *p*-values. A lower number of permutations (30,000) was used for Ferreira *et al*.^37^] dataset, which was also sufficient. The permutation distribution acts as the null distribution from which we empirically compute the p-value for a pathway.

After this, multiple testing correction is performed on the *p*-values using the Benjamini–Hochberg procedure^70^.

### The combined method

We compare our personalised pathway enrichment results with two standard approaches. In the first comparison, we imitate the standard approach of performing DE analysis at the population level and pathway analysis to act as a comparison with our personalised approach. We pool the gene expressions from all the cases and all the controls to obtain a single case-control set of readings, and then compute a single list of differentially expressed features. In this combined method, we again fit two different models (i.e., the *separate* and *joint* models). In case of probe-set data, the DE probe-sets are mapped to their corresponding gene names. To evaluate the enrichment of each pathway, we perform one-sided Fisher’s exact test and compute *p*-values^71^.

In the second comparison, we compare our personalised approach to the results published in Kallionpää et al.^31^ that correspond to our TC, WSC, and WT1D analyses. Briefly, Kallionpää et al.^31^ used the rank-product method^43^ to identify DEGs. The rank-product algorithm is a rank-statistics based technique for identifying DEGs, where a truly significant gene is expected to appear at the top of independently ranked lists of genes per replicate experiment (e.g., per case) in increasing or decreasing order and score a small geometric mean rank. It is a technique derived from biological reasoning. However, it does not account for the heterogeneity of the disease and it is not suitable for the dynamic analysis of time-course data. For TC analysis, expression values were first normalised for each case-control pair using the z-score and case-wise minimum, as well as maximum values are used to identify downregulated or upregulated features. For time-window analyses, in each window (WSC or WT1D), per feature fold changes between cases and matched controls were calculated using linear inter-/extrapolation and then used for rank-product analysis. See Kallionpää et al.^31^ for further details. In order to keep the pathway-level results from Kallionpää et al.^31^ and our approaches comparable, we performed one-sided Fisher’s exact test on the gene-level results from all three analyses presented in Kallionpää et al.^31^ using the pathway information from MSigDB^25,38^.

### Robustness analysis of the Gaussian process models

We performed multiple analyses to demonstrate the robustness of our personalised approach. Detailed methodology and results of these analyses are given in the Supplementary Methods. Briefly, we performed a leave-one-out cross validation analysis to show robustness and efficiency of our GP model in estimating the dynamics of time-course data and predicting unobserved values, i.e., time-course behaviour. In another analysis, we added noise to *Dataset 1* and performed pathway-level inference on the noisy data to demonstrate the robustness of our method to noisy data (noise was added to the original gene expression data, which already contained noise (Supplementary Fig. 5), thus making the generated data even noisier than the original data). By performing correlation tests of the results from these analyses to those from the original analyses, we established our approach to be robust.

### Computational complexity

Time complexity of GP modelling scales as *O*(*N*^3^), where *N* is the number of time points for a probe-set (for a single case-control pair). This is usually non-problematic as most time-course gene expression datasets have small sample sizes.

Our personalised approach largely takes ~3 h to calculate the differential expression scores for all the probe-sets and ~8 h to generate the permutation distribution. Further details can be found in the Supplementary Methods.

### Reporting summary

Further information on research design is available in the Nature Research Reporting Summary linked to this article.

## Supplementary Information

**Supplementary Information**

**Supplementary Data**

**Supplementary Information**

## Data Availability

The datasets analysed in the current study were published by Kallionpää et al.^31^ and Ferreira et al.^37^. Kallionpää et al.^31^ datasets are available in Gene Expression Omnibus (GEO) repository under the accession code: GSE30211; and Ferreira et al.^37^ dataset is available in ArrayExpress under accession code: E-MTAB-1724. No datasets were generated during the current study.

## Code availability

The software implementation of our personalised approach can be found at: https://github.com/SidRama/GP-individual
