# Supplementary material for: A personalised approach for identifying disease-relevant pathways in heterogeneous diseases
Source: NPJ Syst Biol Appl. 2020 Jun 9;6:17. doi: 10.1038/s41540-020-0130-3 (PMC7283216; doi:10.1038/s41540-020-0130-3)
Supplement: Supplementary file 1 — Supplementary Information [file 41540_2020_130_MOESM1_ESM.pdf]

A personalised approach for identifying disease-relevant pathways in heterogeneous diseases

Supplementary

Juhi Somani[1*†], Siddharth Ramchandran[1*†] and Harri Lähdesmäki[1*]

[1]Department of Computer Science, Aalto University, 02150 Espoo, Finland
*e-mail: juhi.somani@aalto.fi, siddharth.ramchandran@aalto.fi, harri.lahdesmaki@aalto.fi
†equal contribution

Supplementary Notes

Datasets from Kallionpää *et al.* [2014]

In this study, we analysed two T1D time-course gene expression datasets (published by Kallionpää *et al.* [2014]) that were generated from the venous blood of the T1D susceptible participants of the DIPP study [Kupila *et al.*, 2001]. Briefly, the total whole-blood RNA was extracted from each sample and hybridised on Affymterix U219 array plates. For a paired case-control experimental design and analysis, Kallionpää *et al.* [2014] decided the pairing of cases and controls in a highly controlled and strict manner, which adhered to the common practices of case-control matching in T1D studies. They matched each seroconverted child (case) to a persistently autoantibody-negative child (control) based on similar date and place of birth, gender, and HLA-DQB1-conferred genetic risk class, which controlled for the confounding effects of these factors. These are some of the most common confounding factors in T1D data that many T1D studies opt to control for in their analyses [Lee *et al.*, 2018; Siemiatycki *et al.*, 1989; Knip *et al.*, 2017; Knip and Simell, 2012; Knip *et al.*, 2017; Dahlqvist *et al.*, 2017]. Matching cases and controls in this manner minimises the effect of these confounding factors on the differential gene expression results and allows us to investigate why certain T1D susceptible children progress to autoantibody positivity and clinical T1D, while their "matching" counterparts do not. To minimise batch effects in case-control comparisons, Kallionpää *et al.* [2014] hybridised the samples from matched case-control pairs on the same plate. Moreover, blood samples taken around the same times in case-control pairs were chosen for hybridisation. Therefore, in our analyses, we maintained the case-control pairing identified by Kallionpää *et al.* [2014]. The raw data (accession code: GSE30211) was downloaded from the GEO database and preprocessed using *affy*-package in *R*. Robust multiarray averaging (RMA) normalisation technique was applied on all the downloaded samples. The case-control pair numbers were kept the same as in Kallionpää *et al.* [2014] for both datasets for comparability. Detailed explanation on sample collection can be found in Kallionpää *et al.* [2014].

Dataset from Ferreira *et al.* [2014]

For assessing generalisability, we performed time-course analysis using our personalised approach on a different T1D dataset comprising of infant samples published by Ferreira *et al.* [2014]. Using this dataset, we aimed to identify disrupted pathways during the early progression of T1D, which is one of the analyses performed on **Dataset 1** from Kallionpää *et al.* [2014]. Specifically, we analysed time-course data from 18 T1D susceptible participants (9 cases and 9 controls) of the BABYDIET study [Hummel *et al.*, 2011]. Ferreira *et al.* [2014] published data from 109 participants of the BABYDIET study of which 87 were autoantibody negative (Aab-) and 22 were autoantibody positive (Aab+). From the 22 Aab+ individuals, we chose 9 individuals as cases who were sampled before and after serconversion, and were sampled at least at three time points each. As per the reasoning provided for Kallionpää *et al.*

[2014] dataset, for each case we found a matched control from the 87 Aab- individuals based on their time of birth, gender and sampling ages. Matching could not be done based on HLA-DQB1-conferred genetic risk class as well, since that information was not available for this dataset. Total mRNA was extracted from the peripheral blood mononuclear cells (PBMCs) isolated from venous blood samples taken at each time point from each individual and hybridised on Affymetrix Human Gene 1.1 ST exon arrays. The raw data (accession code: E-MTAB-1724) was downloaded from ArrayExpress and preprocessed using *oligo*-package in *R*. RMA normalisation was performed at a transcript level using only the core probe-sets. At the transcript level, information is abstracted away from the probe-set level into something that more resembles genes. Transcripts were then annotated with gene names using Ingenuity Pathway Analysis (IPA) before differential expression analysis.

Supplementary Methods

Robustness analysis of the Gaussian process models

Gaussian processes (GPs) are known to robustly estimate missing or unobserved values as they provide confidence intervals along the estimated curves of gene expression [Kalaitzis and Lawrence, 2011]. For GP regression with the Gaussian likelihood model, the predictive distribution is also Gaussian, which provides a point prediction through its mean and uncertainty quantification through its associated variance. GPs can model the true underlying signal while being robust to outliers as well as noisy training data. We demonstrate the effectiveness of our GP modelling in predicting unobserved values (and hence the time-course behaviour) and robustly estimating the dynamics of time-course data in the presence of missing data points. We also demonstrate the robustness of our personalised approach to noise in the data while inferring disrupted pathways.

Leave-one-out cross-validation analysis

In order to illustrate that our GP model can robustly model the dynamics of the time-course data and effectively predict the time-course behaviour, we performed a leave-one-out cross-validation on our GP model fitting using data from case-control Pair 9. Concretely, we randomly removed one data point (either from the case or control) for each probe-set of Pair 9 in **Dataset 1**. We then performed time-course analysis by fitting the GP models and computing the BF-scores as described in Section 3.4. Subsequently, we compared the BF-scores obtained from leave-one-out analysis to the the BF-scores obtained for case-control Pair 9 from the original analysis by computing Pearson's linear and Spearman's rank correlation values, which were 0.9712 and 0.9398, respectively. Originally, 747 genes (after mapping probe-sets to genes) were found differentially expressed (DE) (BF-score > 4) for this pair, whereas after the leave-one-out analysis, 739 genes were found DE of which 91% overlapped with the original list. This shows that the list of DE genes were similar even after randomly removing one data point from the analysis of each probe-set and demonstrates the robustness of our GP model in inferring the dynamics of the gene expression time course data.

Furthermore, we used the estimated gene expression models (i.e. fitted GP model) to predict the randomly removed data point. In other words, we used the better fitting GP model (i.e. *separate* or *joint* model) depending on the computed BF-score to infer whether the original value lies in the 95% confidence interval inferred by the predictive distribution of the GP model. This can be seen as a form of cross-validation. From this analysis, we found that the original expression values of 87% of the randomly removed data points were in the inferred 95% confidence interval of the predictive distribution.

Measurement noise analysis

We simulated the effect of additional measurement noise in the gene expression data of **Dataset 1** by adding random noise sampled from $\mathcal{N}(0, \sigma^2)$ to each probe-set expression measurement from all time points of the 6 case-control pairs, i.e. additive random noise was sampled (and added) for each value in the whole data matrix. After adding the noise, we computed the BF-scores for each probe-set and case-control pair as in Section 3.4 and determined the significance levels of each pathway using our personalised approach for time-course analysis as in Section 3.6. We chose three different variance values,

σ^2, for the noise distribution motivated by the variance observed in the data. In the original gene expression data, approximately 99.7% of the probe-sets had sample variance less than 1 across all time point measurements from all case-control pairs. Supplementary Figure 5 shows a histogram of the estimated probe-set sample variances that are below 1. We performed three separate pathway-level analyses using σ^2 values of 0.04, 0.09 and 0.25 for the noise distribution, showed in red lines in Supplementary Figure 5. These σ^2 values were chosen strategically to represent a range of noise distributions. In Dataset 1, noise variances of 0.04, 0.09 and 0.25 correspond to 12%, 57% and 95% quantiles, respectively.

After performing the pathway analyses on each noisy dataset, we compared the FDR values of all pathways obtained after adding each level of noise with different σ^2 value, to the original FDR values of the pathways using Spearman's rank correlation test in order to understand the effect of adding noise to the gene expression data. We found that the Spearman's rank correlation values were between 0.31 and 0.37, which were found to be highly statistically significant with p-value ≈ 0 (for all three added noise levels). Additionally, we calculated the Spearman's rank correlation on a shorter list of 32 disease-relevant pathways that were found enriched in the original TC analysis (highlighted in colours in Supplementary table 1). The rank correlation values were between 0.58 and 0.77 (decreased as added noise distribution's variance increased), which were also found highly statistically significant with p-values $< 10^{-3}$. Most importantly, many relevant pathways, such as those related to T1D, antigen processing and presentation, interferon gamma signalling, etc. were found significant even after adding noise with $\sigma^2 = 0.25$. These results confirm our methods robustness to added measurement noise.

Computational complexity

Inference for GPs involves the inversion of the kernel matrix. Hence, the time complexity scales as $O(N^3)$, where N is the number of time points for a probe-set. This does not pose a problem in our setting (and for most time-course gene expression datasets) as the number of measured time points for a probe-set per case-control pair is usually quite small. However, a significant amount of research has already been done to bring down the time complexity of GPs. Some popular approaches are the inducing point methods [Snelson and Ghahramani, 2006; Quinonero-Candela *et al.*, 2007; Hensman *et al.*, 2013], structure exploiting methods such as Kronecker or Toeplitz methods [Saatçi, 2012; Cunningham *et al.*, 2008], and even a unifying framework called structured kernel interpolation (SKI) [Wilson and Nickisch, 2015]. Our proposed personalised approach can be trivially adapted in the GP modelling stage to accommodate these methods for scenarios where a large number of data points are present. However, in scenarios where the number of data points is not very large (i.e. the time complexity is not a significant problem) these approximations are unnecessary.

We ran our method on a compute node with an Intel Xeon X5650 2.67 GHz processor. In terms of compute time, our method requires ∼3 hours to calculate the differential expression scores for all the probe-sets and ∼8 hours to generate the permutation distribution for pathway-level inference. Rest of the computations are trivial and do not take a significant amount of compute time. The amount of computation time required can be significantly brought down by reducing the number of pathway permutations (in our case 100,000).

Supplementary Figures

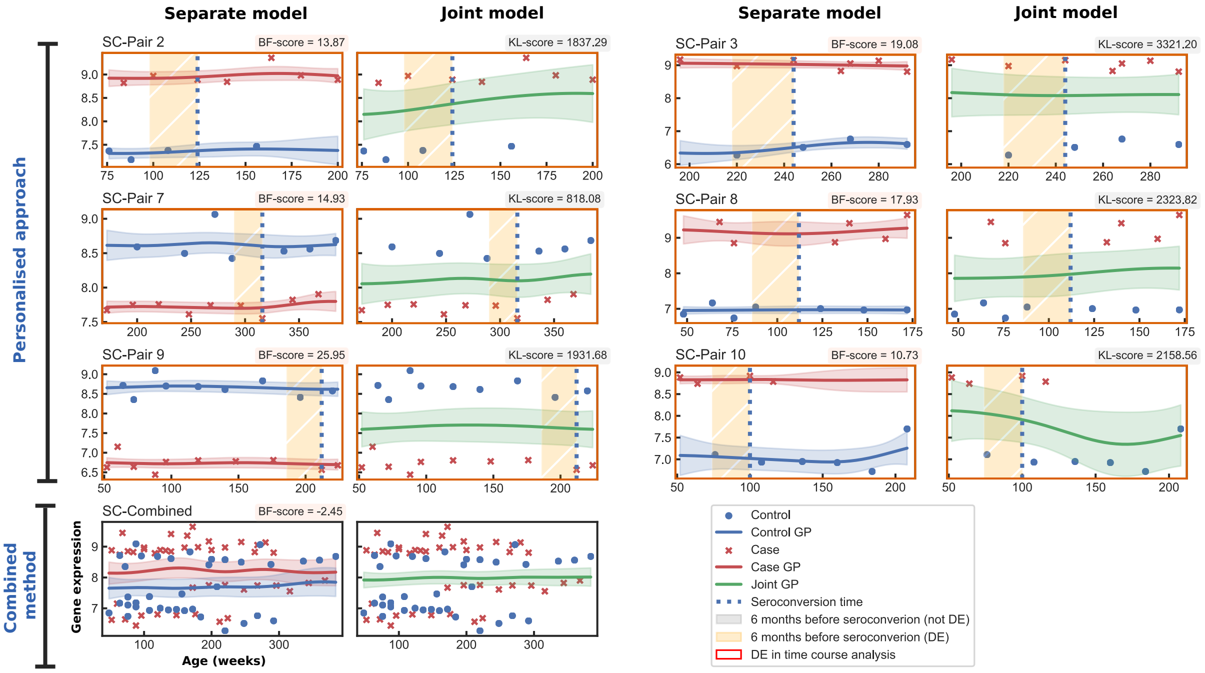

Supplementary Figure 1: Gene expression plots for the *IRF5* gene, visualising the GP model fittings of the separate and joint models for the six case-control pairs from **Dataset 1**. A red border around a plot signifies differential expression (DE) in the time-course analysis and an orange shaded window signifies DE in the time-window analysis. Here, pairs from **Dataset 1** are prefixed with 'SC-'. All profiles belong to the same probe-set as all pairs, including the combined method, identified the same probe-set to have the largest BF-score for *IRF5*.

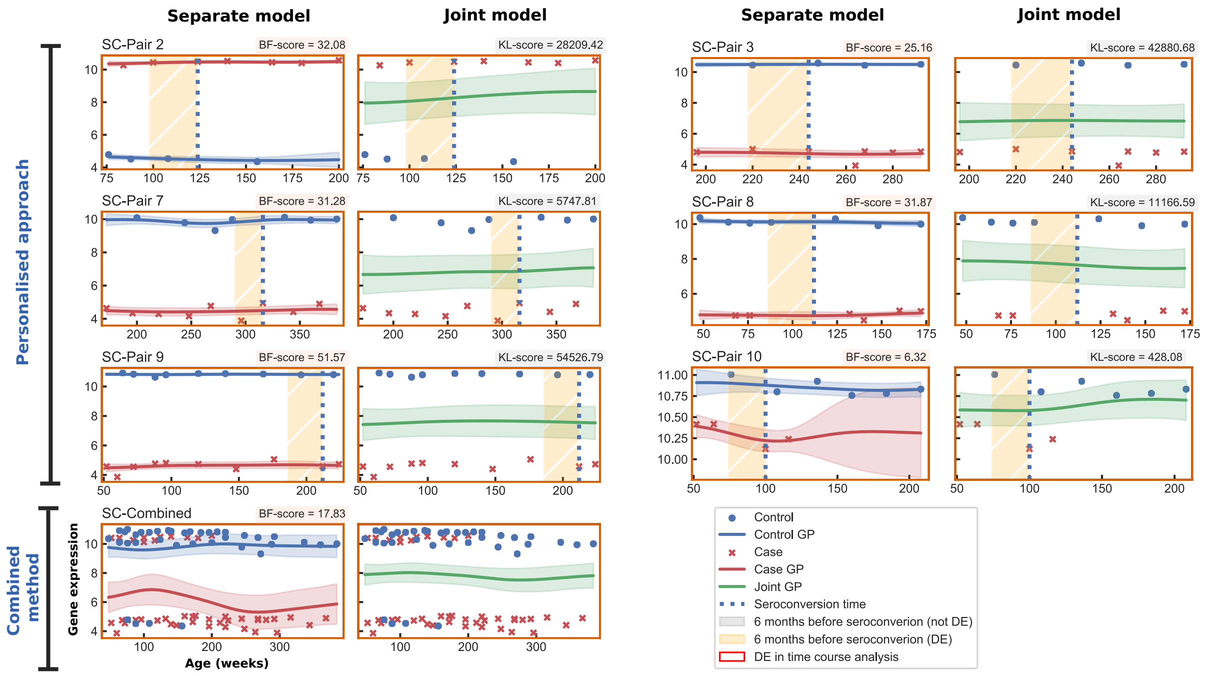

Supplementary Figure 2: Gene expression plots for the *HLA-DPB1* gene, visualising the GP model fittings of the separate model and joint model for the six case-control pairs from **Dataset 1**. A red border around a plot signifies differential expression (DE) in the time-course analysis and an orange shaded window signifies DE in the window analysis. Here, pairs from **Dataset 1** are prefixed with ‘SC-’. All the visualisations belong to the same probe-set, including in the combined method.

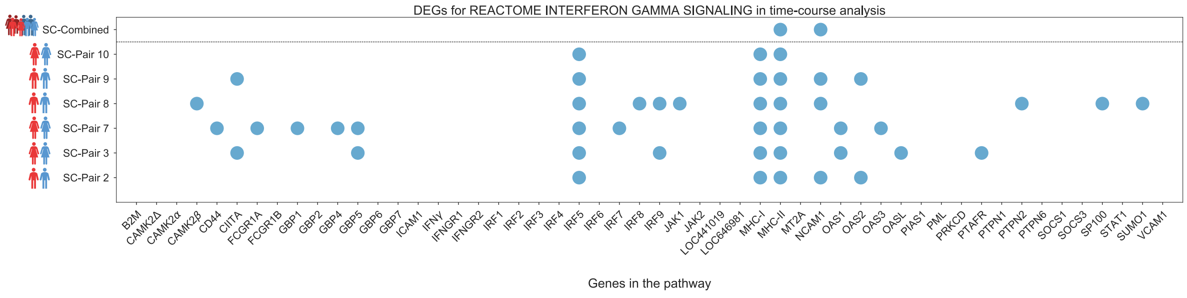

(a)

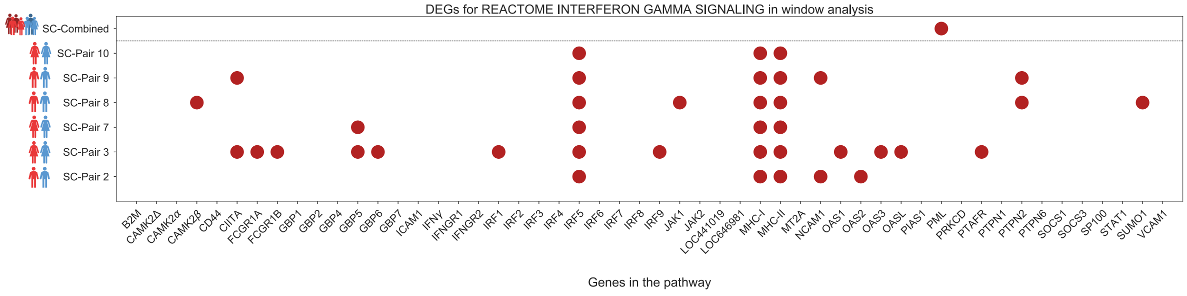

(b)

Supplementary Figure 3: A comparative visualisation of the DEGs between the two approaches for the reactome interferon gamma signalling pathway using **Dataset 1**, prefixed with 'SC-': (a) TC analysis and (b) WSC analysis. A coloured dot signifies that the gene is DE in the corresponding case-control pair. MHC classes I and II are independently marked as DE for each case-control pair if at least one probe-set in the respective group of HLA genes is detected as differentially expressed.

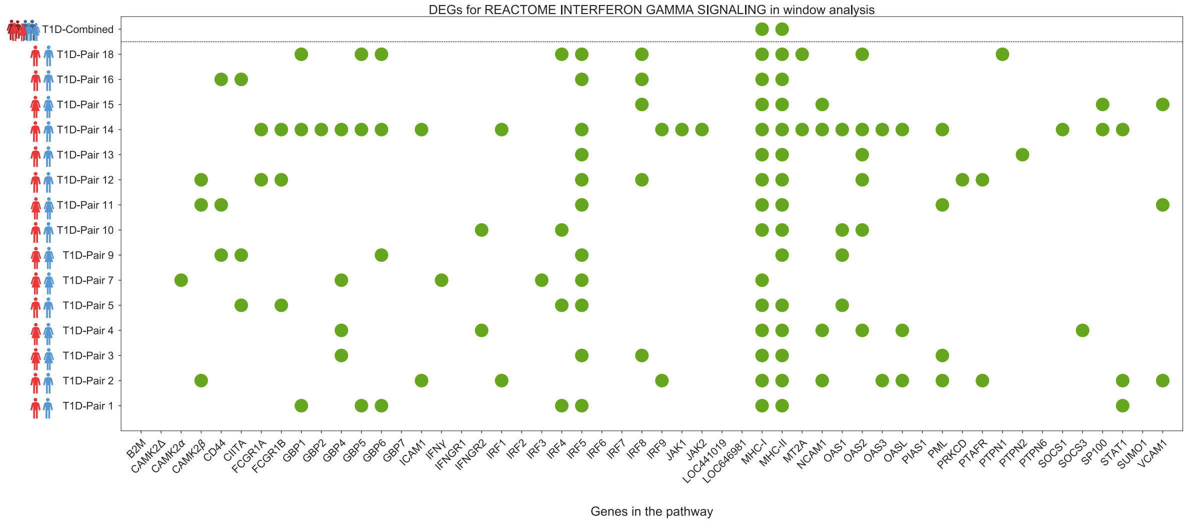

Supplementary Figure 4: A comparative visualisation of the DEGs between the two approaches for the reactome interferon gamma signalling pathway pathway using **Dataset 2**, prefixed with 'T1D-', in the WT1D analysis. A coloured dot signifies that the gene is DE in the corresponding case-control pair. MHC classes I and II are independently marked as differentially expressed for each case-control pair if at least one gene in the respective group of HLA genes is detected as differentially expressed.

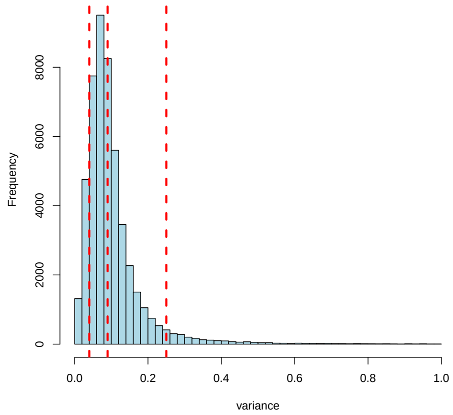

Supplementary Figure 5: Sample variances of 99.7% of the probe-sets that are calculated across all samples from the 6 case-control pairs of **Dataset 1**. Red line shows the σ^2 values chosen for the distribution $\mathcal{N}(0, \sigma^2)$ from which additive random noise was sampled for each probe-set expression measurement from each individual in **Dataset 1**.

References

Cunningham, J. P., Shenoy, K. V., and Sahani, M. (2008). Fast gaussian process methods for point process intensity estimation. In *Proceedings of the 25th international conference on Machine learning*, pages 192–199. ACM.

Dahlqvist, S., Rosengren, A., Gudbjörnsdottir, S., Pivodic, A., Wedel, H., Kosiborod, M., Svensson, A.-M., and Lind, M. (2017). Risk of atrial fibrillation in people with type 1 diabetes compared with matched controls from the general population: a prospective case-control study. *The Lancet Diabetes & Endocrinology*, **5**(10), 799–807.

Ferreira, R. C., Guo, H., Coulson, R. M., Smyth, D. J., Pekalski, M. L., Burren, O. S., Cutler, A. J., Doecke, J. D., Flint, S., McKinney, E. F., *et al.* (2014). A type i interferon transcriptional signature precedes autoimmunity in children genetically at risk for type 1 diabetes. *Diabetes*, **63**(7), 2538–2550.

Hensman, J., Fusi, N., and Lawrence, N. D. (2013). Gaussian processes for big data. *arXiv preprint arXiv:1309.6835*.

Hummel, S., Pflüger, M., Hummel, M., Bonifacio, E., and Ziegler, A.-G. (2011). Primary dietary intervention study to reduce the risk of islet autoimmunity in children at increased risk for type 1 diabetes: the babydiet study. *Diabetes care*, **34**(6), 1301–1305.

Kalaitzis, A. A. and Lawrence, N. D. (2011). A simple approach to ranking differentially expressed gene expression time courses through gaussian process regression. *BMC bioinformatics*, **12**(1), 180.

Kallionpää, H., Elo, L. L., Laajala, E., Mykkänen, J., Ricaño-Ponce, I., Vaarma, M., Laajala, T. D., Hyöty, H., Ilonen, J., Veijola, R., *et al.* (2014). Innate immune activity is detected prior to seroconversion in children with hla-conferred type 1 diabetes susceptibility. *Diabetes*, page DB_131775.

Knip, M. and Simell, O. (2012). Environmental triggers of type 1 diabetes. *Csh Perspect Med*, **2**(7), a007690.

Knip, M., Luopajärvi, K., and Härkönen, T. (2017). Early life origin of type 1 diabetes. In *Seminars in immunopathology*, pages 653–667. Springer.

Kupila, A., Muona, P., Simell, T., Arvilommi, P., Savolainen, H., Hämäläinen, A.-M., Korhonen, S., Kimpimäki, T., Sjöroos, M., Ilonen, J., *et al.* (2001). Feasibility of genetic and immunological prediction of type i diabetes in a population-based birth cohort. *Diabetologia*, **44**(3), 290–297.

Lee, J. J., Thompson, M. J., Usher-Smith, J. A., Koshiaris, C., and Van den Bruel, A. (2018). Opportunities for earlier diagnosis of type 1 diabetes in children: A case-control study using routinely collected primary care records. *Primary care diabetes*, **12**(3), 254–264.

Quinonero-Candela, J., Rasmussen, C. E., and Williams, C. K. (2007). Approximation methods for gaussian process regression. *Large-scale kernel machines*, pages 203–224.

Saatçi, Y. (2012). *Scalable inference for structured Gaussian process models*. Ph.D. thesis, Citeseer.

Siemiatycki, J., Colle, E., Campbell, S., Dewar, R. A., and Belmonte, M. M. (1989). Case-control study of iddm. *Diabetes Care*, **12**(3), 209–216.

Snelson, E. and Ghahramani, Z. (2006). Sparse gaussian processes using pseudo-inputs. In *Advances in neural information processing systems*, pages 1257–1264.

Wilson, A. and Nickisch, H. (2015). Kernel interpolation for scalable structured gaussian processes (kiss-gp). In *International Conference on Machine Learning*, pages 1775–1784.
